# Preliminary Investigation on Hygienic-Sanitary Quality of Food Vending Machines

**DOI:** 10.3390/ijerph20085557

**Published:** 2023-04-18

**Authors:** Giuseppina Caggiano, Vincenzo Marcotrigiano, Marilena D’Ambrosio, Piersaverio Marzocca, Valentina Spagnuolo, Fabrizio Fasano, Giusy Diella, Anna Paola Leone, Marco Lopuzzo, Domenico Pio Sorrenti, Giovanni Trifone Sorrenti, Maria Teresa Montagna

**Affiliations:** 1 Interdisciplinary Department of Medicine, University of Bari Aldo Moro, 70124 Bari, Italy; 2 Prevention Department, Food Hygiene and Nutrition Service, Local Health Authority BT Barletta-Andria-Trani, 76125 Trani, Italy; 3 Department of Precision and Regenerative Medicine and Ionian Area, University of Bari Aldo Moro, 70121 Bari, Italy; 4 Freelance Biologist, Microbiological Laboratory, 73100 Lecce, Italy

**Keywords:** coffee, microbial contamination, public health, vending machine

## Abstract

The consumption of hot drinks dispensed from vending machines has become a common practice, both in workplaces and during free time. Every day, millions of bulk drinks are sold, but the quality of the products distributed may not always be guaranteed, as it is related to many factors such as the quality of the water, the raw materials used, and the effectiveness of the equipment’s cleaning system. The purpose of this study is to evaluate the hygienic-sanitary requirements of hot drinks and vending machine surfaces. The investigation highlighted the microbial contamination of both coffee and vending machine surfaces. Although the ”coffee break” is usually considered a moment of pleasure, apparently not subject to specific jurisdiction, the products dispensed can represent a health risk if the hygienic conditions are not fully respected. Therefore, official controls carried out by the Prevention Department represent a suitable way for evaluating and guaranteeing the hygienic-sanitary requirements, providing for corrective actions, when needed, to protect consumers.

## 1. Introduction

Despite technological and scientific progress, infectious diseases are still a major problem in terms of social and health impact [1,2]. To date, infectious risk is a problem both in the healthcare sector and in any situation where human activities are carried out, due to the environmental exposition to potential infectious agents [3]. 

People’s habits have definitely changed, as they spend many hours a day in community living environments, increasing the chances of being exposed to pathogenic microorganisms. Increasingly often, we consume fleeting and quick preparation meals, using ready-to-use foods able to satisfy any gastronomic need [4]. Therefore, the consumption of hot drinks sold from automatic machines has become a common practice, especially in leisure and in workplaces such as hospitals, schools, libraries, municipalities, and shopping centers. It is a sharing moment intended as a coffee break among colleagues.

A food vending machine (FVM) is an equipment providing products and/or services at the request of a user, subject to a payment. The first FVM was created by Heron of Alexandria, a Greek mathematician and engineer (I–II century AD). He built a coin-operated holy water dispenser, placed in front of the temples to avoid crowds and confusion. 

An FVM was imported into Italy in the 1950s, and over the years, it has reached greater visibility both through a more refined and accurate image of distributors, and the increasingly huge variety of products offered. The first espresso coffee dispenser was realized during early 1960s. It was able to grind coffee and dispense coffee through the insertion of an ITL 50 coin. Even today, after more than 60 years, all drinks are produced from soluble and lyophilized powder, mixed with water in a mixer once the beverage is selected, while coffees are generally produced from coffee beans ground and pressed on a filter where the hot water passes through the coffee powder [5].

Every day, millions of drinks are sold, but the quality of the products distributed is not always guaranteed, depending on various factors: the quality of the raw materials used, the water quality intended for human consumption, the temperature (which should not be lower than +80 °C), the microbial load of the powders (inside of which, after the heat drying process, fungi, bacteria, and bacterial spores can survive), and the effectiveness of the cleaning and disinfection procedures. In compliance with EU legislation on food safety [6], for the vending distribution of food and beverages, it is essential to carry out a specific food risk assessment, putting in place adequate procedures to guarantee consumers a safe product [7]. Therefore, all the FVMs’ Food Business Operators (FBOs) must provide an adequate and accurate plan of cleaning and sanitizing procedures for the internal and external surfaces of the machines, included in the Food Safety Management System, which is useful to reduce the contamination risk to acceptable levels [8].

In the Apulia Region, similarly to other areas where Multi-Annual Control Plans are applicable, local planning provides a specific assignment for food matrices and analytical parameters to be investigated, divided into microbiological, chemical and physical parameters. As a rule, sampling performed on FVMs is not part of the activities of the Regional Integrated Control Plans. The aim of this study was to evaluate the hygienic and sanitary features of unpackaged hot drinks and, simultaneously, of vending machine surfaces. 

## 2. Materials and Methods

### 2.1. Study Design

In the period September–October 2022, 50 FVMs, belonging to different companies, were randomly selected in the territory of a province of the Apulia Region (Southern Italy). They were primarily chosen considering their location in contexts such as schools, hospitals, bus and railway stations, and municipal public institutions. Alternatively, the selection of the 24 h cafeteria services (i.e., a vending machine shop available 24 h a day to offer customers snacks, drinks, and coffee) was made favoring the road location and easy accessibility for users. The FVM companies in the area investigated were previously informed of this activity in written form, through a simple request to participate in the study, aimed at investigating the hygienic-sanitary characteristics of vending machines. The study was characterized by the sampling of hot coffee and internal and external surfaces in the area in which the beverages are dispensed. 

### 2.2. Coffee and Surface Sampling

**Figure 1:**
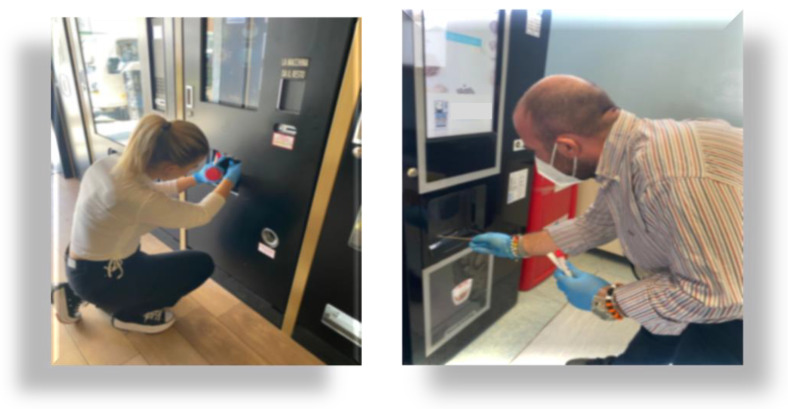
Healthcare professionals sampling internal and external surfaces of FVMs.

Since the identified surfaces to be sampled were of modest size, the sampling was performed using sterile swabs moistened with 10 mL of sterile neutralizing solution (ESC swab Neutralizing Rinse Solution, Liofilchem Diagnostic Srl, Roseto degli Abruzzi, Teramo, Italy), as shown in Figure 1.

For each FVM, three surfaces were examined: the plastic window used by users to take the disposable coffee cup, the inside part of the dispensing compartment, and the beverage-dispensing nozzle. In parallel, 5 mL of unsweetened coffee was also sampled. As soon as the coffee was dispensed in the cup, the temperature was measured by means of a probe thermometer (XS Instruments ITA, Carpi, Italy).

A total of 140 samples (50 coffees without sugar and 90 surface swabs) were subjected to microbiological investigation, searching for bacteria and fungi.

### 2.3. Microbiological Investigations

The swabs were suspended in 10 mL of neutralizing solution, and 1 mL was plated in duplicate on Plate Count Agar (PCA; Becton-Dickinson, Heidelberg, Germany) for bacterial counts, and on plates with Sabouraud dextrose agar with chloramphenicol (0.05%) (Biorad, Marnes-La-Coquette, France) for mycological count (molds and yeasts). Two milliliters of each coffee sample were plated on PCA agar and Sabouraud dextrose agar to evaluate the bacterial and fungi load, respectively. 

In addition, 1 mL of each swab and coffee sample was seeded on Wurtz agar plates (Wurtz Lactose Agar–Liofilchem) and Sabouraud dextrose agar with chloramphenicol (0.05%) (Biorad, Marnes-La-Coquette, France) for the isolation and identification of strains. 

After incubation at 30 ± 1 °C for 72 h for the detection of bacteria and at 28 ± 1 °C for 8 days for the detection of fungi, the results were expressed as colony-forming units/mL (cfu/mL) for coffee and as cfu/swab for surface investigations.

Bacteria and yeasts were identified with appropriate biochemical tests using the Biolog automated system (Rigel Process and Lab, Motta Visconti, Milan, Italy). Filamentous fungi were identified by evaluating the standard cultural characteristics (i.e., morphology, colony color, type of surface growth, and macro- and microscopic examination) as described by de Hoog et al. [9].

Two-by-two comparisons were made with the Chi-square test or Fisher’s exact test, taking into consideration the results of bacteria and fungi, with respect to the types of swabs performed (beverages, internal swabs, and external swabs). The Shapiro–Wilk normality test was used to verify the normal distribution of the bacterial and fungal load. The distribution was considered normal if *p* > 0.05. The Kruskal–Wallis rank sum test with post hoc Dunn’s test (Benjamini–Hochberg test method) and Wilcoxon rank sum test were used to compare the results of the bacterial and fungal load, with respect to the types of swabs performed (beverages, internal swabs, and external swabs). Tests with *p* < 0.05 were considered statistically significant. R version 3.6.1 was used to perform all statistical tests.

## 3. Results

Overall, 74.3% of the samples were contaminated, 65.7% with bacteria and 32.9% with fungi. Particularly, 25 of the 50 coffee samples (50%) were contaminated with bacteria only. The mean temperature of the coffee samples was +54.5 °C (range +49/+63 °C).

Comparing the data obtained on the coffee with those obtained on the surfaces with the Chi-square test or Fisher’s exact test, the coffee samples were less contaminated by bacteria than the internal surface swabs (50% vs. 73.2%—*p* = 0.024) and the external surface swabs (50% vs. 75.5%—*p* = 0.008). There was no significant difference comparing the total bacterial load of the external versus internal swabs (75.5% vs. 73.2%). 

The distribution of the fungal and bacterial load was not normal (Shapiro–Wilk normality test—bacteria W = 0.55057, *p* < 0.001—fungi W = 0.5335, *p* < 0.001), so the non–parametric Kruskal–Wallis rank sum test with post hoc Dunn’s test (Benjamini–Hochberg test method) and Wilcoxon rank sum test (W) was used for the comparison between the load groups. 

The Kruskal–Wallis rank sum test reported significant results (Kruskal–Wallis Chi-squared = 26.081, *p* < 0.001). In particular, the external surfaces (median bacterial load 21 cfu/swab—range 1–300) were more contaminated than the coffee (median bacterial load 1 cfu/mL—range 1–110) and the internal surface swabs (median bacterial load 8 cfu/swab—range 1–300), and the internal surfaces were more contaminated than the coffee. These differences are statistically significant, as reported in the post hoc Dunn’s test (Benjamini–Hochberg test method) results (external surface vs. coffee: *p* < 0.001, external surface vs. internal surface: *p* = 0.01, internal surface vs. coffee: *p* = 0.003). The most frequently isolated bacteria were *Stenotrophomonas maltophilia*, *Enterobacter cloacae*, *Klebsiella pneumoniae*, and *Staphylococcus* spp.

In regard to the search for fungi, the presence of filamentous fungi (*Aspergillus fumigatus*, *Aspergillus niger*, *Penicillium* spp., *Cladosporium* spp., and *Neurospora crassa*) and yeasts (*Candida parapsilosis*, *Torulaspora globose*, and *Clavispora lusitaniae*) was detected in 32.9% of samples (40.8% external and 63.4% internal swabs). The coffee samples were always negative for fungi. No statistically significant difference emerged from the comparisons between the fungal load in the external surfaces (median fungal load 5.5 cfu/swab—range 1–300) and in the internal surfaces (median fungal load 1 cfu/swab—range 1–300) (W = 174, *p* = 0.1174). The external surface swabs were more frequently contaminated with bacteria than fungi (75.5% vs. 40.8%—*p* = 0.0005). 

**Figure 2:**
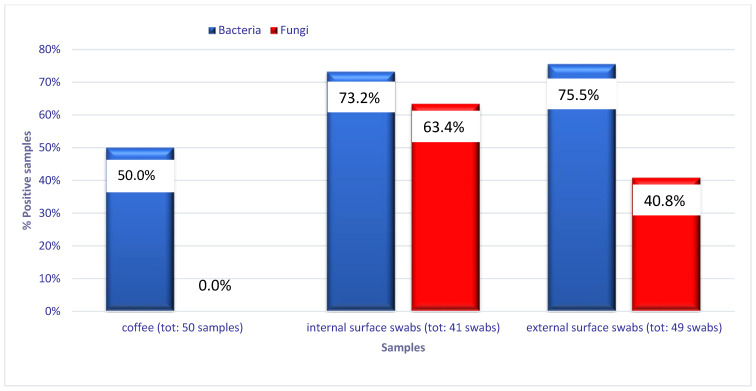
Positive samples (%) of coffee and internal/external surfaces of FVMs.

No significant difference was detected between bacterial and fungal contamination (73.2% vs. 63.4%) on the internal surfaces, while fungal contamination was statistically significant when comparing internal versus external surface swabs (63.4% vs. 40.8%, *p* = 0.032), as shown in Figure 2.

With regard to the comparison between FVM with open and closed beverage dispensing flaps, a non-statistically significant result was observed.

## 4. Discussion

Since FVMs were imported into Italy in the 1950s [10], they have spread widely, now located in indoor community environments and also along the streets, such as in 24 h cafeteria services, becoming a perfect opportunity for the consumer who can benefit from them at any time. Today, FVMs are well equipped and supply a wide range of food products; however, if the hygienic conditions are not adequately respected, consumers could be exposed to the risk of contracting foodborne diseases. Indeed, according to Regulation (EC) no. 852/2004, to ensure the correct application of the HACCP system, the same FBO, as the manager of the FVMs, must provide the cleaning and sanitization of the equipment, establishing the execution of hygiene procedures, considering that the frequency is related to the type of machine, the frequency of use, and the location [6].

According to the handbook of good hygiene practice for automatic food distribution, edited by the Italian association of vending machines and approved by the Italian Ministry of Health, the water supply, the management of the raw materials, and the related frequency of the cleaning and sanitation of the equipment represent the main aspects to be assessed and monitored to ensure the dispensation of safe food and drinks. Furthermore, particular attention must be paid regarding food defense aspects, which can be evaluated within the food safety management system, especially regarding prepacked foods [8].

This study highlighted bacterial and fungal contamination, especially on the internal and external surfaces of FVMs, though less frequent in coffee, probably because it is a hot drink. The temperature detected on our coffee samples was between +50 °C and +60 °C, despite the Italian guidelines suggesting temperatures above +80 °C. Other authors have also reported temperatures of about +65/+70 °C [8].

It is known that contact between drinks and surfaces of a food machine, or previous inadequate maneuvers in drink dispensing, as well as the nozzle and the external part of the dispenser itself, can favor the proliferation of bacterial and fungal species, representing a serious risk for public health [5,11]. 

The most frequently isolated bacteria were Gram-negative, in particular *Enterobacteria* on external surfaces, which are reasonably related to contact with consumers’ hands. 

The possibility of contamination related to contact with the consumers’ hands could also suggest the presence of other microorganisms not investigated in this preliminary study, such as emerging foodborne pathogens that need specific microbiological techniques for identification, or gastrointestinal and respiratory pathogenic viruses, which are easily transmissible through the hands. Indeed, the experience of the COVID-19 pandemic has taught us the importance of hands as a vehicle for the transmission of infectious diseases and, consequently, of implementing hand sanitization procedures. Therefore, it could be appropriate to place dispensers around FVMs, such as hydroalcoholic solution points, and to inform the consumers of the importance of the disinfection treatment while using vending machines. 

To the best of our knowledge, this is the first study that evaluated the microbial contamination of both internal and external surfaces of coffee vending machines in Italy. Several studies around the world have been conducted on coffee contamination only, focusing on the search for chemical parameters [12,13].

Traversa et al. evaluated the presence of the emerging foodborne pathogen *Arcobacter* spp. in raw milk vending machines, suggesting the need to enlarge the analytical investigations to other microorganisms not yet included in the food safety criteria [14].

Regarding the mycological investigation, filamentous fungi prevail, mostly belonging to the genus *Aspergillus* and *Penicillium*. The presence of mold may not be related to environmental contamination only, but to freeze-dried products, used as raw materials in FVMs. 

The fungal contamination of the internal surfaces was statistically significant compared to the external ones. The contamination could derive not only from the incorrect management and cleaning of the internal components of the FVM compared to the external ones, but also from the raw materials and ingredients. Coffee is susceptible to contamination, especially by fungi, so this food matrix is included in the list of regulated foods because of mycotoxin contamination and, specifically, by Ochratoxin A (OTA), in accordance with Reg. (EC) 1881/2006 and subsequent amendments [15]. In general, mycotoxins are a product of the secondary metabolism of some molds. In our case, *Aspergillus* spp. and *Penicillium* spp. were frequently found: it seems useful to highlight that these genera are the most capable fungi in producing mycotoxins. Although OTA is predominantly produced by *Aspergillus ochraceus* and *Penicillium viridicatum*, recent research has demonstrated that some black Aspergilli species, including *A. niger*, are sources of OTA in food products [16], and *A. niger* was one of the most frequently isolated in our study. Moreover, even if our coffee samples were negative for fungi, it is known that the absence of molds is not a guarantee for the absence of mycotoxins too. Studies have demonstrated respiratory abnormalities among workers in coffee roasting and packaging plants due to contamination of the environment and coffee samples by molds, including toxigenic species [17]; this suggests that the impact of the fungal contamination of coffee cannot be underestimated.

Furthermore, the role of water should not be overlooked, as it could come from the public water network or be directly supplied by FBOs or maintainers, always deposited in tanks. Then, the water passes through descaling filters, in which fungal spores thicken, facilitating their circulation in the FVM’s circuit.

The most abundant fungus isolated from the internal surfaces is probably related to a more humid internal environment. Humidity is an ideal condition for fungal proliferation, largely demonstrated both in foods [18,19,20,21] and indoor environments [22,23,24,25]. These data suggest the need to improve the safety of FVMs by applying proper maintenance, cleaning, and sanitization interventions. Furthermore, the presence of filamentous fungi, as well as yeasts isolated from FVMs in the hospital, highlights the risk of healthcare-associated infections.

In compliance with EU legislation on food safety, the automatic vending carried out through FVMs—unpackaged or prepacked—is included among the activities subject to communication through the submission of a Certified Notification of Commencement of Business (SCIA) through the Unique Branches of Productive Activities (SUAP), managed by the municipal administrations; for these activities, it is also essential to carry out a specific food risk assessment, in accordance with Regulation (EC) no. 852/2004 [6]. Accordingly, the official control on food safety must represent an opportunity to deeply evaluate, through health professionals belonging to a multidisciplinary and adequately trained team, the requirements and contribute to the adoption of corrective actions, where necessary [26,27,28]. In these activities, very different from the usual retail trade activities manned in a fixed position by personnel, the risk assessment process must focus on many aspects, including: the place where the FVM is located; the training of the FBOs; the expiry date of the raw materials; the maintenance of the cold chain in the case of the storage of perishable foods; the connection of the FVM to the public water supply network or the presence of a water storage tank, if the system provides for the preparation of loose drinks, including instant drinks; and the methods and frequency of cleaning and sanitizing operations.

The water used in food businesses, which includes trade activities carried out through vending machines, must be guaranteed with regard to both microbiological and chemical parameters. The presence of any critical point in the distribution network (filters, softeners, resins, osmosis systems, etc.), often present in FVMs, must be carefully assessed through a risk-based approach. In our country, a recent Legislative Decree which will enter the legislation at the end of March 2023, adopting the Community Directive 2020/2184, defines both treated and untreated water as “water intended for human consumption”, promoting the guarantee of compliance with the parameter values up to the point of use, as well as through the drafting of Water Safety Plans [29,30].

The Good Hygiene Practices manuals, validated by the Italian Ministry of Health, can guide the risk assessment process supporting the FBOs, as well as provide assistance for the competent authorities, to better investigate specific control phases [8]. The risk perception of these activities is generally underestimated, as the “coffee break” is usually considered a convivial moment, synonymous with healthiness, apparently not subject to specific jurisdiction. From a legal viewpoint, being considered food businesses for all intents and purposes, these installations are under the community sector legislation, which provides for specific obligations, from notification to the drafting of the Hazard Analysis and Critical Control Point manual (even if simplified) and the FBO’s own checks, up to official controls. 

To the best of our knowledge, this is the first study that investigates the hygienic-sanitary characteristics of FVMs in the Apulia Region, simultaneously evaluating the microbiological aspects of the dispensed beverages and adjacent surfaces.

Given the absence of personnel involved in the management and replenishment of the FMVs, it was not possible to acquire information on the frequency and methods of cleaning and sanitization normally adopted. Therefore, we believe it is necessary to deeply investigate this topic in order to intervene in any critical management issues. 

## 5. Conclusions

Food and drinks purchased through self-service shops represent a large part of local trade, therefore being worthy of targeted controls. This type of official controls are not always easy to perform, since they are not food businesses manned by staff. The outcome of our study highlights some critical hygienic-sanitary issues that must be considered and investigated, even in the case of foodborne illnesses and in related epidemiological investigations. Important efforts are required, both from FVM managers to implement procedures aimed at guaranteeing adequate hygienic quality and from healthcare personnel in charge of official controls on food safety, to inspect these situations with greater awareness of all the aspects to be considered. 

Focusing to new perspectives, future studies could aim to research the emerging foodborne pathogens, viruses not only of gastrointestinal origin, and mycotoxins on the surfaces and within the coffee drinks of FVMs, in order to better understand the customers’ risk of exposure to microbiological contaminants through one of the food and beverage service dispensers currently most chosen by consumers.
